# Extended self-efficacy theory to explain patients’ non-adherence to tuberculosis treatment in Mpumalanga healthcare facilities

**DOI:** 10.4102/curationis.v49i1.2840

**Published:** 2026-10-26

**Authors:** Harma Motsi, Bester Chimbo

**Affiliations:** 1 School of Computing, College of Science, Engineering and Technology, University of South Africa, Pretoria, South Africa

**Keywords:** tuberculosis, patient, adherence, treatment, nurses, pill burden, patient satisfaction

## Abstract

**Background:**

Tuberculosis (TB) remains a major burden in the province of Mpumalanga, South Africa. Treatment adherence is a key factor in the outcome of therapy.

**Objectives:**

To develop a conceptual framework based on extended self-efficacy theory to explain patients’ non-adherence to TB treatment in healthcare facilities in the province of Mpumalanga.

**Method:**

The study employed a quantitative approach with a cross-sectional survey design. Data were collected through structured questionnaires distributed to 89 patients receiving TB treatment. Descriptive statistics, the Pearson correlation coefficient and multiple regression analysis were among the many analyses carried out in this study.

**Results:**

Self-efficacy has a significant influence on medication literacy (*β* = 0.136, *t* = 2.359, *p* < 0.001), patient satisfaction (*β* = 0.105, *t* = 2.115, *p* < 0.001) and pill burden (*β* = 0.166, *t* = 2.977, *p* < 0.001) towards non-adherence to TB treatment and, thus supporting hypotheses H1, H2 and H3. There was a negative relationship between patient satisfaction and attitudes toward healthcare workers (*β* = 0.039, *t* = 0.684, and *p* > 0.72). Therefore, H4 is not supported. Attitude towards TB treatment has a significant effect on patient satisfaction towards non-adherence to TB treatment (*β* = 0.143, *t* = 2.691, *p* < 0.001); hence, H5 was accepted.

**Conclusion:**

Self-efficacy had a partial significant mediating effect on the relationship between medication literacy and medication adherence. This study emphasises self-efficacy and the importance of improving treatment attitudes, medication literacy and the efforts of healthcare professionals to prevent non-adherence to TB treatments.

**Contribution:**

The findings contribute to the body of knowledge and offer a platform for assessing the current strategies on fighting the disease through the developed research model aimed at determining the obstacles to non-adherence to TB treatments at healthcare facilities. The research model suggests various strategies that ultimately reduce the rate of treatment default, limits community transmission, and lower the global risk of multi-drug-resistant tuberculosis (MDR-TB).

## Introduction

### Background

Tuberculosis (TB) is an infectious disease that remains the leading cause of death among adults worldwide, as reported by the World Health Organization (WHO 2025). The WHO’s 2022 TB report states that there are an estimated 10.6 million TB patients worldwide, 1.4 million TB-related fatalities among HIV-negative individuals and an additional 187 000 deaths among HIV-positive individuals. Africa accounted for 25% of all cases, with Asia coming in second at 44% (WHO 2022). In addition, the burden of drug-resistant TB as a percentage of the number of TB infections continues to impact public health (Tiberi et al. 2022). It is also expected that 21% of newly diagnosed and previously treated cases of TB harbour drug-resistant tuberculosis (DR-TB), which is believed to be responsible for an estimated 182 000 deaths yearly (WHO 2023). Hong et al. (2020) found that 44% of patients in an extensive study in South Africa had to change their drug schedule because they had clinically proven hearing loss. In total, 52% of people with multidrug-resistant tuberculosis (MDR-TB) were depressed, according to a recent review of published studies (Duko, Bedaso & Ayano 2020).

In South Africa, the prevalence of TB is unevenly distributed, with some regions accounting for over 70% of cases. Since 2018, Mpumalanga has recorded a rise in positive TB cases (Mpumalanga Provincial Government [MPG] 2021). One of the municipalities in the nation with the highest rate of TB is Ehlanzeni District Municipality in Mpumalanga; since 2018, there have been over 1000 deaths and nearly 13 000 infections (Kalamane 2021). According to the South African National Institute for Communicable Diseases (2018), the province was recently placed sixth for having a high incidence rate of TB (402 per 100 000). Furthermore, Mpumalanga was recently found to have one of the highest rates of TB that is resistant to rifampicin, an antibiotic medication used to treat TB (Mashamba et al. 2022). Mpumalanga province has the highest HIV prevalence of any province in 2022, with an estimated 890 000 individuals living there (17.4%) (Mabena 2024). Meanwhile, the simultaneous presence of both TB and HIV-TB co-infection poses distinct challenges since both diseases have a mutual connection that leads to significant negative consequences (Olivier & Luies 2023). In addition, Olivier and Luies (2023) stated that the co-infection of HIV-TB and TB poses particular problems because of the reciprocal link between the two illnesses, which can have detrimental consequences.

In addition, early detection and treatment of TB are considered the most critical interventions to prevent the development of DR-TB. Treatment for TB is crucial to reducing transmission and preventing drug resistance in a timely and efficient manner (Oliveira et al. 2021). Tuberculosis treatment is carried out with treatment that requires a long time (at least 6–9 months) consistently (Saukkonen et al. 2025). Apart from physical factors, psychological and mental factors also need to be considered in TB treatment, namely individual understanding, which can influence perceptions of the disease and the treatment process. Self-efficacy in the effectiveness of treatment to cure TB is very important (Kurniyawan et al. 2022). In this case, self-efficacy refers to a person’s ability to make decisions that show effort in achieving progress and show resilience in completing various life tasks (Luszczynska & Schwarzer 2005; Schwarzer & Jerusalem 1995). Using the extended self-efficacy theory (ESET), this study aims to determine the factors impacting patients’ non-adherence to TB treatment in Mpumalanga healthcare facilities. The findings of the study will assist healthcare professionals in creating targeted treatments that can increase these individuals’ drug compliance and improve treatment results.

## Research model and hypotheses development

### Behavioural theories of non-adherence and related studies

Numerous behavioural theories have been used to develop tools to increase adherence in chronic illnesses (Patton et al. 2017). The Health Belief Model (HBM) (Tarkang & Zotor 2015) and Theory of Planned Behaviour (TPB) (Baudouin, Wongsawat & Sudnongbua 2020) are two health psychology models that can be used to identify the root reasons for unhealthy behaviour related to halting the spread of disease infections. Theory of Planned Behaviour is an extension of the Theory of Reasoned Action (TRA). Although TRA states that a person’s intention towards behaviour is produced by two basic aspects, namely attitude towards behaviour and subjective norms (Fishbein & Ajzen 2011), perceived behavioural control (Ajzen 1991) is an extra feature in TPB. Health Belief Model is a theoretical model that explains the process of transformation and its relationship to health behaviour, according to Tarkang and Zotor (2015). It will produce an assessment of a person’s ability and motivation to change their state of health. The Reasoned Action Approach (RAA) and Social Cognitive Theory (SCT; ed. Bandura 1986, 2001; Cameron, Fleszar-Pavlovic & Khachikian 2020) are the two other main theories. Theory of Reasoned Action (Azjen & Schmidt 2020; Fishbein & Ajzen 2011) and TPB (Azjen & Schmidt 2020; Fishbein & Ajzen 2011) were its antecedents. Both have been effective in forecasting behaviour and recommending areas for intervention.

Bogg and Milad (2020) discovered that adherence to TB rules was linked to attitudes, self-efficacy, subjective norms and intentions using these theories. The study by Mohammadi et al. mentions the connection between self-efficacy and sickness perception. Nurses can assist patients in this area by implementing treatments to enhance disease perception and self-efficacy, according to the study’s researchers, who acknowledge that patients with poor self-control abilities should have their illness perception assessed (Mohammadi et al. 2022). The purpose of this study was to develop a conceptual framework based on Extended self-efficacy theory (ESET) to explain why patients in Mpumalanga province’s healthcare institutions do not stick to their TB treatment.

### Research model

The behavioural theories: Health Belief Model (HBM); Theory of Planned Behavior (TPB), Socioemotional Selectivity Theory (SST); Social Cognitive Theory (SCT); Reasoned Action Approach (RAA), and Social Cognitive Theory (SCT), which were previously discussed in the section on behavioural theories of non-adherence and related studies, are essential for creating customised behavioural interventions to address patients’ unique medication adherence obstacles. If any of the aforementioned behavioural theories were used, they would still be applicable to the research. The study’s objective was to develop a conceptual framework based on ESET to explain patients’ non-adherence to TB treatment in medical facilities in the province of Mpumalanga. Hence, self-efficacy theory (SET), which was chosen as the study’s guiding theory. Furthermore, studies have demonstrated that self-efficacy can support individuals in maintaining self-management when they make behavioural changes, such as reducing alcohol consumption (Mellentin et al. 2020) or stopping smoking (Krauthammer et al. 2020). Therefore, in order to add to the body of knowledge, SET was adopted and was expanded by adding additional constructs to examine the factors impacting TB patients’ non-adherence to medicine.

For the purpose of this current study, self-efficacy is defined by the five constructs of Albert Bandura’s SET: verbal persuasion, mental and physiological states, mastery experiences, and vicarious experiences. It was said that these factors were modestly to strongly linked to self-efficacy (Litalien, Morin & McCoach 2023). The personality trait of self-efficacy is an important part of SCT (Bandura 1977), which shows how self-efficacy can greatly affect how people think, feel and act (Dinh & Bonner 2023). Self-efficacy (SLE), which can be seen in Figure 1, was adopted as the dependent variable in this study. According to Puente-Fernández et al. (2020), failing can make a person feel less confident in their own abilities and often leads to avoiding the task at hand. As previously stated, the primary objective of this study was to develop a conceptual framework that would be evaluated and validated in order to comprehend why some patients in Mpumalanga healthcare facilities do not follow their TB treatment regimen. To better represent the objectives of the study, the four SET constructs – medication literacy (MEL), pill burden (PB), patient satisfaction (PST) and attitude towards tuberculosis treatment (ATBT) were renamed. In addition, a new construct, attitude towards healthcare workers (AHW), was added to SET, and Figure 1 shows the study framework that was developed.

**FIGURE 1:**
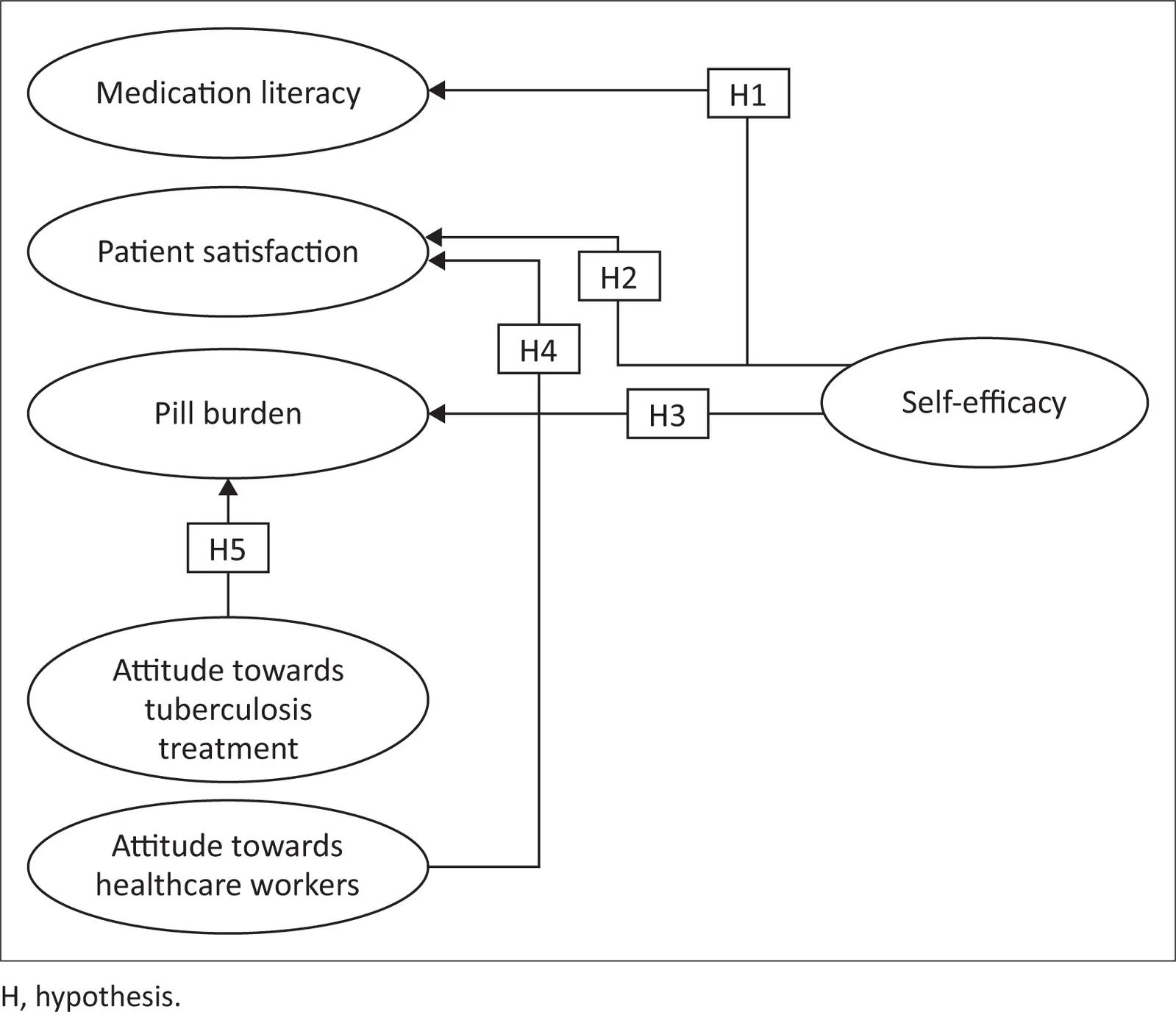
Research model.

### Variables description of self-efficacy theory and hypotheses development

This section presents the dependent variables and independent factors for each construct, along with a definition, related literature and a hypothetical suggestion.

#### Self-efficacy

In 2020, Gutierrez and Sakulbumrungsil released a systematic review study that looked at the factors that affect Filipinos with high blood pressure taking their medications as prescribed. According to the study’s results, not taking medications as prescribed can be caused by a number of things, including being young, single, poorly educated, not knowing much about the disease, not having many friends or family who support you, not believing in your own abilities, and not believing that you can control your illness (Gutierrez & Sakulbumrungsil 2021). Low self-efficacy and a negative view of one’s disease can make it harder to take medications as prescribed (Gutierrez & Sakulbumrungsil 2021). As for people with chronic diseases, how they see their sickness can affect their sense of self-efficacy (Mohammadi et al. 2022). Given that SLE pertains to health literacy in the context of medication use, it is reasonable to assume that it may have a major impact on the link between ML, PD, PS and ATBT. Therefore, the following theories are proposed to explore the relationship between SLE and ML, PS and PB:

**H1:** There is a significant and positive relationship between self-efficacy and medication literacy.

**H2:** There is a significant and positive relationship between self-efficacy and patient satisfaction.

**H3:** There is a significant and positive relationship between self-efficacy and pill burden.

#### Attitude towards healthcare workers

Studying patients’ thoughts and feelings is very important on a global level because they have a big impact on how healthcare systems around the world keep getting better (Al-Jabri et al. 2021). Clinical competence is only one part of quality of care; non-clinical factors of healthcare are also very important to service quality (Stavropoulou et al. 2022). For this reason, patient accounts of respect tend to focus on dignity more than liberty (Fernandez et al. 2024) because patients’ experiences of respect and disrespect are mostly relationship-based. From the patients’ point of view, rudeness can mean being ignored, treated badly or looked down upon (Entwistle, Cribb & Mitchell 2024). Patients who are satisfied with their care will follow treatment instructions more closely, have a good relationship with their doctors, and get better care in the long run (Doyle, Lennox & Bell 2013; Sim et al. 2021). To examine this relationship, the following hypothesis is proposed:

**H4:** There is a significant and positive relationship between attitude towards healthcare workers and patient satisfaction.

#### Attitude towards tuberculosis treatment

Knowledge of the disease is linked to having a better approach towards TB (Bashorun et al. 2020). Knowledge of TB signs and how to avoid getting it usually leads to better attitudes and more frequent visits to the doctor, which speeds up the diagnosis of TB and makes it easier for people to stick with treatment (Fana, Ijeoma & Sotana 2019). Bashorun et al. (2020) indicated that the goals of TB control must be met if the public is properly informed, feels and acts about TB prevention, treatment and control. News about health care can get to a lot of people quickly and help them learn more (Eibich & Goldzahl 2020). According to Dilas et al. (2023), a patient’s motivation, which comes from knowing about the disease, having supportive family members and having help from their healthcare provider, is the most important thing that keeps them following their treatment plans. To examine this relationship, the following hypothesis is proposed:

**H5:** There is a significant and positive relationship between attitude towards tuberculosis treatment and pill burden.

## Research methods and design

### Research design, study population and design

The constructs of the ESET were adopted and investigated in this study using a quantitative cross-sectional survey design. Self-efficacy and medication beliefs are known factors that affect treatment adherence. This research design collects the data required to answer general questions about a particular group (Cox & Fell 2020). The researcher used a cross-sectional survey design to collect the data. According to Rothman et al. (2021), a cross-sectional study can be useful in identifying the need for services, illustrating the extent of an issue in a community, or characterising the burden of a condition within a group. Then, as needed by a cross-sectional survey design, the individuals’ information was collected once. This study used a self-administered questionnaire as its sole method for collecting quantitative data.

### Study setting and the target population

Five public TB healthcare facilities that were considered reference Directly Observed Treatment, Short course (DOTS), tertiary and regional facilities served as the study’s sites. These included the TB units that provide patients with TB screening, diagnosis and treatment services at Bongani Hospital (BHTPU), Kabokweni Clinic (KCTBU), Kanyamazane Clinic (KACTBU), Lydenburg Gate Clinic (LGCTBU) and Matsulu Community Health Clinic (MCHCTBU). According to Schneider and Iacobucci (2020) and Sekaran and Bougie (2019), the target audience needs to be specified in terms of time, space and other factors. The study focused on TB patients who were specifically chosen for these medical facilities.

#### Inclusion criteria

All TB patients who are undergoing anti-TB treatment medications, whether in the intensive or continuation phase, have been enrolled within the last 6 months, are older than 18, and have given their consent to be included in the study.

#### Exclusion criteria

Patients with TB who failed to provide informed consent or who were gravely unwell at the time of data collection were not included in this study.

### Sampling techniques and sample size

#### Sampling technique

Van Haute (2021) states that sampling is the process of choosing a smaller group from a bigger one that is representative of the whole. Studying the sample will help us figure out what the results mean for the whole community. People who are close when the researcher is collecting data can be chosen for the sample using convenience sampling, which is also known as accidental sampling (Basti & Madadizadeh 2021). For the initial outreach to participants in the study, the researcher employed the convenience sampling strategy, drawing on the literature mentioned above. The proportions of patients sampled per each tuberculosis healthcare facility is displayed in Table 1.

**TABLE 1:** Proportions of patients sampled per each tuberculosis healthcare facility (*N* = 84).

Name of the TB healthcare facility	Province	Patients selected (*n*)
BHTPU	Mpumalanga	19
KCTBU	Mpumalanga	13
KACTBU	Mpumalanga	18
LGCTBU	Mpumalanga	15
CHCTBU	Mpumalanga	14

TB, tuberculosis; BHTPU, Bongani Hospital; KCTBU, Kabokweni Clinic; KACTBU, Kanyamazane Clinic; LGCTBU, Lydenburg Gate Clinic; MCHCTBU, Matsulu Community Health Clinic.

#### Sample size

As Bolarinwa (2020) states, a sample size is the number of people the researcher picks to take part in the study. Effect size (*f*^2^) = 0.25, alpha = 0.05 and power = 0.95 were the parameters used by G*Power program to determine the sample size; based on these values, the total sample size would be 84 participants. In order to identify any changes in the sample, an effect size of 0.25 is regarded as conservative. One hundred questionnaires were distributed to TB units at BHTPU, KCTBU, KACTBU, LGCTBU and MCHCTBU as part of this study. Of these, 84 were completed and deemed valid for analysis, representing a 70% response rate.

### Data collection

Thus, a quantitative approach was used with a survey questionnaire comprising five sections. Section A consists of the demographic background information of the participants, while Section B consists of the independent variables: MEL, PST, PB, ATBT, AHW and self-efficacy (SEL). Medication literacy, PST, PB, AHW, PST and SEL all had six items, while AHW had four variable items based on the research model in Figure 1. Thus, items were examined with the support of the 5-item scale ranging from 1 = strongly disagree to 5 = strongly agree as the instrument for data collection. A self-administered questionnaire was used to collect data for the study, which used a quantitative research approach. From a total of 100 distributed questionnaires, a total of 92 were returned, and 84 were suitable for analysis. It was initially prepared in English and then translated into Xitsonga. Pretesting of the questionnaires was carried out at Ogies Clinic TB Clinic (OCTBC) in Mpumalanga. The TB clinic was chosen for pretesting because it has a high number of registered TB patients. Twenty-three respondents (10%) of the sample size were used for pretesting. The feedback from 20 respondents of the pretest was used to modify the data collection instrument.

### Data analysis

The statistical analysis was carried out using the Statistical Package for the Social Sciences version 30.0 (SPSS; IBM Corporation 2025). Widely used in quantitative research, SPSS is a well-known software program that provides a variety of analysis choices, such as data transformations and many output formats. Descriptive statistics, the Pearson correlation coefficient and multiple regression analysis were among the many analyses carried out in this study.

### Ethical considerations

The ethical approval for the study was granted by the University of South Africa-CAES Health Research Ethics Committee (Ref 2020/CAES_HREC/002). Every technique was used in compliance with the applicable rules and regulations. A written informed consent form was obtained from each participant prior to enrolment. In order to maintain the strictest confidentiality, identifiable information was kept anonymous during data analysis, including survey replies and personal information.

## Results

### Socio-demographic characteristics of the respondents

Eighty-four participants made up the study sample, providing a fairly even distribution of ages. Regarding gender, there were 29 (34.5%) females and 55 (65.5%) males. Of them, 27 (32.1%) were between the ages of 38 and 47, 24 (28.5%) were between the ages of 28 and 37, 10 (11.9%) were under the age of 27, and 10 (11.9%) were beyond the age of 58. Table 2 provides an overview of their demographic attributes.

**TABLE 2:** Sociodemographic characteristics of the tuberculosis patients from Bongani Hospital, Kabokweni Clinic, Kanyamazane Clinic, Lydenburg Gate Clinic and Matsulu Community Health Clinic (*N* = 84).

Variable	Sub-variable	Frequency	%
Gender	Male	55	65.5
	Female	29	34.5
Age group (years)	≤ 27	10	11.9
	28–37	24	28.6
	38–47	27	32.1
	48–57	13	15.5
	≥ 58	10	11.9
Marital status	Married	17	20.2
	Single	58	69.0
	Divorced or widowed	9	10.9
Level of education	No formal education	46	47.6
	Primary	9	10.7
	Secondary	24	28.6
	Tertiary	11	13.1
Occupation	Employed	15	17.9
	Self employed	38	45.2
	Unemployed	31	36.9

### Descriptive statistics and correlation analysis

An examination of consistency was conducted in order to evaluate the dependability of each individual variable: MEL, PST, PB, ATBT and AHW and SEL. The results showed that the reliability of all the variables did not exceed the acceptable threshold value of 0.70 (SEL = 0.96, PS = 0.92, PAT = 0.93, PB = 0.94 and APHW = 0.96). Self-efficacy and MEL have a somewhat positive correlation (*r* = 0.446; *p* < 0.01). However, Table 3 clearly shows that there is a substantial correlation between SEL and PST (*r* = 0.4415; *p* < 0.01). In addition, there is a positive and strong relationship between SEL and PB (*r* = 0.204; *p* < 0.01). Pill burden and ATBT had a positive and significant correlation (*r* = 0.183; *p* < 0.01), as seen in Table 3. Table 3 shows the summary of the descriptive statistics, Pearson’s correlation and alpha coefficients.

**TABLE 3:** Descriptive statistics, Pearson’s correlations and reliability coefficients among variables.

Variable	Mean	s.d.	1			2			3			4			5			6
			SEL	*r*	*p*	PST	*r*	*p*	MEL	*r*	*p*	PB	*r*	*p*	ATBT	*r*	*p*	
SEL	3.98	0.64	0.96	-	-	-	-	-	-	-	-	-	-	-	-	-	-	-
PST	3.56	0.76	-	0.441*	< 0.01	0.92	-	-	-	-	-	-	-	-	-	-	-	-
MEL	4.17	0.68	-	0.446*	< 0.01	-	0.338*	< 0.01	0.93	-	-	-	-	-	-	-	-	-
PB	2.23	0.8	-	0.204*	< 0.01	-	0.183*	< 0.01	-	0.176*	< 0.01	0.95	-	-	-	-	-	-
ATBT	3.08	0.88	-	0.265*	< 0.01	-	0.189*	< 0.01	-	0.185*	< 0.01	-	0.186*	< 0.01	0.96	-	-	-
AHW	3.54	0.87	-	0.432*	< 0.03	-	0.401*	< 0.01	-	0.182*	< 0.01	-	0.163*	< 0.01	-	0.184*	< 0.01	0.94

s.d., standard deviation; SEL, self-efficacy; PST, patient satisfaction; MEL, medication literacy; PB, pill burden; ATBT, attitude towards tuberculosis treatment; AHW, attitude towards healthcare workers.

* , Pearson correlation analysis *r* values are Pearson correlation coefficients.

To further provide greater legitimacy for the data and the model, measures of composite reliability (CR), discriminant validity and convergent validity for each factor were also used to verify internal consistency (see Table 4). The average variance extracted (AVE) for each loaded construct ranged from 0.543 to 0.744, while the CR for each factor ranged from 0.781 to 0.923, above the cutoff condition (CR > 0.7) (Hu & Bentler 1999). Since it was found to be higher than 0.50, a threshold that is frequently utilised, this demonstrated convergent validity (Fornell & Larcker 1981). The square value of AVE should be greater than the correlation coefficient between the different constructs in the study framework in Figure 1. This is another way to check discriminant validity (Henseler, Ringle & Sarstedt 2015). For discriminant validity to be checked, Fornell and Larcker (1981) state that each number of the square root of AVE had to be greater than all of the inter-factor correlations. In this study, each of the constructs had a significant level of *p* < 0.001.

**TABLE 4:** Composite reliability, convergent and discriminant validity.

Variable	CR	AVE	MSV	MAX (*H*)	PST	SEL	MEL	PD	ATBT	AHW
PST	0.965	0.754	0.231	0.976	0.865	-	-	-	-	-
SEL	0.941	0.638	0.231	0.939	0.479*	0.798	-	-	-	-
MEL	0.953	0.685	0.203	0.935	0.452*	0.369*	0.826	-	-	-
PB	0.934	0.842	0.147	0.986	0.268*	0.198*	0.382*	0.919	-	-
ATBT	0.964	0.808	0.140	0.973	0.461*	0.224*	0.187*	0.189*	0.877	-
AHW	0.942	0.813	0.062	0.975	0.221*	0.189*	0.192*	0.251*	0.246*	0.897

MEL, medication literacy; PST, patient satisfaction; PD, pill burden; ATBT, attitude towards tuberculosis treatment; AHW, attitude towards healthcare workers; CR, composite reliability; AVE, average variance extracted; MSV, maximum shared variance; MAX (*H*), maximal reliability; SEL, self-efficacy.

* , *p* < 0.001.

### Model fit findings

To ascertain the model fit using the corresponding indices, a range of values has been produced using the SPSS AMOS software. Table 5 displays the model fit indices, which were obtained by a confirmatory factor analysis (CFA) based on the exploratory factor analysis (EFA) of the patients’ overall satisfaction. The results show that the following values were obtained: the comparative fit index (CFI) = 0.980, the relative fit index (RFI) = 0.951, the incremental fit index (IFI) = 0.980, the Tucker-Lewis index (TLI) = 0.976, the normed fit index (NFI) = 0.959, and the root mean square error of approximation (RMSEA) = 0.045. In accordance with guidelines (Hu & Bentler 1999; Marsh, Hau & Wen 2004; McNeish, An & Hancock 2018), all of these values are either above or at the recommended levels.

**TABLE 5:** Model fit statistics.

Model	Acceptance level	Model value	Result
Chi square	-	780.547	Satisfactory fit
Degrees of freedom	-	415.000	Satisfactory fit
Chi square/*df*	-	1.881	Satisfactory fit
Standardised root mean residual (SRMR)	< 0.05	0.042	Satisfactory fit
Comparative fit index (CFI)	> 0.95	0.980	Satisfactory fit
Normed fit index (NFI)	> 0.90	0.959	Satisfactory fit
Tucker Lewis index (TLI)	-	0.976	Satisfactory fit
Relative fit index (RFI)	-	0.951	Satisfactory fit
Incremental fit index (IFI)	-	0.980	Satisfactory fit
Root mean square error of approximation (RMSEA)	< 0.05	0.045	Satisfactory fit

*df*, degrees of freedom.

### Confirmatory factor analysis

The CFA confirmed the relationship among the observed variables and their underlying latent variables. It was used to assess the loadings of items on a particular factor. The values of the factor loadings, while conducting the CFA, are given in Table 6. The statistical values of the CFA all met the criteria standards for the adequacy of fit. The values of factor loadings showed the strength of the relation of the factors with their respective constructs. The maximum value for factor loading is 1. The highest factor loading value in this model was 0.925, and the lowest value was 0.709. All the values of factor loading confirmed the validation of the construct validity, which was measured by inspecting the loadings of the items and their reliability.

**TABLE 6:** Results of the confirmatory factor analysis.

Constructs	Cronbach alpha	Construct items	Factor loadings
Medication literacy (MEL)	0.968	MEL1	0.789
		MEL2	0.817
		MEL3	0.878
		MEL4	0.896
		MEL5	0.854
		MEL6	0.752
Pill burden (PB)	0.942	PB1	0.783
		PB2	0.825
		PB3	0.863
		PB4	0.786
		PB5	0.858
		PB6	0.882
Attitude towards tuberculosis treatment (ATBT)	0.946	ATBT1	0.857
		ATBT2	0.861
		ATBT3	0.865
		ATBT4	0.789
		ATBT5	0.894
		ATBT6	0.877
Attitude towards healthcare workers (AHW)	0.936	AHW1	0.858
		AHW2	0.866
		AHW3	0.879
		AHW4	0.862
		AHW5	0.783
		AHW6	0.865
Self-efficacy (SEL)	0.974	SEL1	0.786
		SEL2	0.853
		SEL3	0.868
		SEL4	0.864
		SEL5	0.847
		SEL6	0.808
Patient satisfaction (PST)	0.966	PST1	0.930
		PST2	0.935
		PST3	0.887
		PST4	0.873
		PST5	0.951

### Hypothesis testing using a multiple regression analysis

The multiple regression analysis used to identify all of the factors influencing total patient satisfaction in public TB hospitals is displayed in Table 7. The findings showed that the seven independent variables: medication literacy (MEL), PST, PB, ATBT and AHW accounted for 37.2% (adjusted *R*^2^ = 0.367, *F* = 74.33 and *p* = 0.0001) of the variance in the outcome variable, SEL. In order to test for multicollinearity, the tolerance values ranged from 0.766 to 0.900 (a value closer to zero indicates a problem with collinearity), while the variance inflation factor (VIF) ranged from 1.238 to 1.511 (a value greater than three indicates a problem with collinearity). The findings showed that there was no multicollinearity in the data. Two of the four variables were significant and had a positive impact on patient satisfaction, according to the values in Table 7; the other three variables’ values were not significant.

**TABLE 7:** Multiple regression models (dependent variable: self-efficacy).

Variable	*B* coefficient		95% confidence interval for *B*			Collinearity statistics	
	*B*	*T*	Sig	Lower bound	Upper bound	Tolerance	VIF
Constant	-	9.591	0.000	1.318	1.994	-	-
1. Medication literacy (MEL)	0.136	2.359	0.000	0.224	0.504	0.766	1.306
2. Patient satisfaction (PST)	0.105	2.115	0.000	0.136	0.516	0.856	1.169
3. Pill burden (PB)	0.166	2.977	0.026	0.013	0.249	0.900	1.111
4. Attitude towards tuberculosis treatment (ATBT)	0.039	0.684	0.728	0.000	0.280	0.806	1.241
5. Attitude towards healthcare workers (AHW)	0.143	2.691	0.000	0.147	0.368	0.846	1.154

Note: Model summary *R* = 0.555, *R*^2^ = 0.308, *F* = 49.06, *p* = 0.000, Durbin-Watson (DW) = 2.01. Based on Figure 1, it is hypothesised that: H1: SEL → MEL; H2: SEL → PST; H3: SEL → PB; H4: AHW → PS; H5: ATBT → PST.

VIF, variance inflation factor; Sig, significance.

Self-efficacy has a significant impact on medication literacy (ML) (*β* = 0.136, *t* = 2.359, *p* < 0.001), PST (*β* = 0.105, *t* = 2.115, *p* < 0.001) and pill burden (PB) (*β* = 0.166, *t* = 2.977, *p* < 0.001) towards non-adherence to TB treatment, supporting hypotheses H1, H2 and H3 as shown in Table 7. There was a negative correlation between AHW and patient satisfaction (PST) (*β* = 0.039, *t* = 0.684, and *p* > 0.72). Therefore, H4 is not supported. Subsequently, the findings demonstrated that patient satisfaction (PS) with non-adherence to TB treatment is significantly impacted by ATBT (*β* = 0.143, *t* = 2.691, *p* < 0.001), leading to the acceptance of H5.

## Discussion

This section addresses complex interactions between variables in the ESET and thoroughly examines the theoretical underpinnings of study hypotheses (H1 – H5), explaining how outcomes agree with or differ from hypotheses. Medication literacy, PST, PB, ATBT and AHW were among these variables. Based on actual findings, theories derived from the body of existing literature are also examined. The study’s findings are synthesised with previous research, implications are discussed, recommendations are framed within the hypothesis, and the study’s contributions to improving respondents’ knowledge and attitudes can influence their actions in preventing TB transmission.

### Hypothesis 1: There is a significant and positive relationship between self-efficacy and medication literacy

There was a positive and statistically significant correlation between SEL and MEL (*β* = 0.136, *t* = 2.359, *p* < 0.001). H1 is supported as a result. Self-efficacy and MEL are significantly positively correlated. There is a moderately favourable correlation (*r* = 0.446; *p* < 0.01) between SEL and MEL. This relationship illustrates how SEL and MEL are interdependent. According to Table 2, 13.1% of the patients had higher education, 28.6% had secondary education, and 10.7% had primary education. As a result, almost half of the patients have a formal education. Higher education has a favourable effect on self-efficacy with relation to medication adherence, which means that people with higher levels of education are typically more confident in their ability to manage their prescription regimens.

According to AbuAlreesh and Alburikan (2019), people with low health literacy were more likely to misinterpret information on medicine labels and were less likely to actively discuss drug information with physicians and participate in drug decision-making. One could speculate that this finding is related to educating patients with pulmonary TB, which could influence how serious their condition is viewed and function as a formative component in motivating them to take their medication more regularly.

### Hypothesis 2: There is a significant and positive relationship between self-efficacy and patient satisfaction

The correlation (*β* = 0.105, *t* = 2.115, *p* < 0.001) between SEL and PST. As a result, H2 is supported. The findings demonstrate a significant positive relationship between self-efficacy and patient satisfaction. Nonetheless, Table 3 makes it very evident that SEL and PST are positively and significantly related (*r* = 0.4415; *p* < 0.01). It makes sense when one considers that most people’s motivation to take medication ultimately stems from SEL and PST. Medication views are associated with patients’ perceptions of the advantages and disadvantages of suggested therapies, as well as their necessity and concerns (Cruz-Jesus, Pinheiro & Oliveira 2019). This result is in line with previous studies that showed self-efficacy was one of the elements influencing medication adherence in patients with chronic illnesses, also supporting our findings (Kim et al. 2021). The results of the study are in line with previous research by Lu et al. (2020), which discovered that patient satisfaction with healthcare services, health literacy and the quality of TB medication all had an impact on non-adherence to treatment. This result is consistent with research by Darvishpour, Mansour-Ghanaei and Mansouri (2022), who found that better medication adherence, better communication with healthcare professionals and active involvement in disease management are all associated with higher levels of self-efficacy.

### Hypothesis 3: There is a significant and positive relationship between self-efficacy and pill burden

The relationship between SEL and PB is positive and statistically significant (*β* = 0.166, *t* = 2.977, *p* < 0.001). In light of that, H3 is supported. The results of the study point to a significant positive relationship between SEL and PB. The correlation between SEL and PB of (*r* = 0.204; *p* < 0.01) is also positive and significant, as shown in Table 3. This result is in line with the conclusion drawn by Stewart, Moon and Horne (2023) that interventions are more successful when patient education materials and communication strategies are adapted to people with different health literacy levels, cultural backgrounds and linguistic preferences. These findings corroborate those of Ampofo, Khan and Ibitoye (2020) about patient education, which guarantees that patients are informed about their diagnosis, available treatments and potential side effects.

These results are also supported by Gutierrez and Sakulbumrungsil (2021), who discovered that people with low self-efficacy and a negative outlook on their disease may find it more difficult to take prescription medications as prescribed. A person’s feeling of self-efficacy may be impacted by how they perceive their chronic condition (Mohammadi et al. 2022). These results are consistent with earlier research by Wang and Shan (2021), which discovered a relationship between self-efficacy and health literacy and noted that those with higher levels of health literacy also had higher levels of self-efficacy (Wang & Shan 2021).

### Attitude towards healthcare workers

#### Hypothesis 4: There is a significant and positive relationship between attitude towards healthcare workers and patient satisfaction

There was a negative, statistically insignificant correlation between AHW and PST (*β* = 0.039, *t* = 0.684, *p* > 0.72). Therefore, H4 is not supported. The findings of the study point to a negative relationship between AHW and PST. 47.6% of patients have no formal education, according to Table 2. Higher education is linked to a better comprehension of treatment regimens, which enhances adherence, according to numerous studies (Berkman et al. 2011; Cutler & Lleras-Muney 2010). These findings imply that perceptions of healthcare providers have little bearing on patient satisfaction. The results run counter to those of Gül, Helvacıoğlu and Saraçlı (2023), who claimed that medical staff service attitudes are often thought to have a substantial impact on patient satisfaction. Long wait times, poor communication, perceived disrespect, or a lack of confidence in the provider’s abilities are common causes of negative attitudes towards healthcare workers, which may explain the statistically insignificant negative association between attitude towards healthcare workers and patient satisfaction, according to the conclusion drawn from these findings.

### Attitude towards tuberculosis treatment

#### Hypothesis 5: Attitude towards tuberculosis treatment and pill burden are positively and statistically significantly correlated

Thus, with *β* = 0.143, *t* = 2.691, and *p* < 0.001, H5 is supported. Table 3 shows Pearson’s correlation of *r* = 0.183, *p* < 0.01 between ATBT and PB. The results also demonstrate that ATBT and PB have a positive, significant relationship between the two variables. These findings are in line with recent research showing that patients may encounter pharmaceutical burden at any stage of their drug journey (Redwood et al. 2024). The intricacy of the medication regimen, financial worries and disturbance in daily life are only a few of the many contributing aspects that make up the multifarious medication burden (Teni et al. 2021).

Male patients who are single have far greater rates of medication non-adherence, especially when they do not have social support. Studies show that single men frequently miss the emotional support network and direct assistance (such as medication reminders) that wives provide (Taylor et al. 2023). The Table 2 data, which indicate that 69.0% of the patients were unmarried, corroborate these conclusions. Educational attainment has a major impact on medication adherence; higher levels of education are often linked to higher adherence rates (Berkman et al. 2011; Ulley et al. 2019). Low levels of education are linked to inadequate understanding of illnesses and treatment plans, which reduces adherence.

## Conclusion

The study effectively addressed a research requirement by using the validated features to develop a theoretical framework for assessing the factors influencing patients’ non-adherence to TB treatment in medical facilities in the province of Mpumalanga. It is crucial to look into the health behaviours of pulmonary TB patients with reference to medication adherence. Based on an ESET, this study sought to find the literature on professional interventions and significant factors for enhancing adherence to pulmonary TB medication. In all, 37.2% of the variance in the outcome variable, SEL, was found to be explained by seven independent factors (adjusted *R*^2^ = 0.367, *F* = 74.33 and *p* < 0.001) as shown in Table 7.

In addition, MEL, PST, PB, ATBT and AHW were among these variables. Five hypotheses’ assumptions generated from the conceptual model in Figure 1 were investigated using regression and correlation analysis. While H4 was rejected, the regression model validated hypotheses H1, H2, H3 and H5. Improving medication literacy and self-efficacy may help improve medication adherence in older patients with multimorbidity, as this population has a high rate of poor drug adherence. As a result, healthcare professionals should understand the need to evaluate and advance drug literacy and put self-efficacy-boosting techniques into practice. The results of the study show that self-efficacy plays a crucial role in MEL, PST, and PB in relation to non-adherence to TB therapy. There are no data that connect professional interventions to significant factors that promote health through life-based adherence.

Furthermore, the study offers valuable insights into barriers to TB treatment adherence, but is limited by its small sample size, single-site design, short period of data collection and reliance on self-reported data. To obtain a better understanding of the variables influencing patients’ satisfaction with hospital care, future research studies may use larger sample sizes and different sampling techniques. Moreover, researchers can endeavour to investigate additional critical factors, such as examining diseases in detail, which can contribute to a deeper knowledge of the components influencing patient satisfaction at the tertiary level of health issues. In addition, future research should include larger, multi-site samples and longitudinal designs to better understand the determinants of non-adherence and inform interventions. Comprehensive treatment programs should be promoted to address the multifactorial determinants of non-adherence to TB treatment. Therefore, to guarantee the best possible medication adherence, it is advised to put in place continual education programmes for patients, especially regarding their beliefs and superstitions. It also urges further investigation into treatment adherence problems and solutions.
